# Leveraging AlphaFold for innovation and sustainable health research in Africa

**DOI:** 10.1038/s41467-025-56545-y

**Published:** 2025-02-04

**Authors:** Emmanuel Nji, Katharina C. Cramer, Nicolas V. Rüffin, Fatoumata G. Fofana, Walid Heiba, Safiétou Sankhe

**Affiliations:** 1 BioStruct-Africa, Huddinge, Sweden; 2 BioStruct-Africa, Kwame Nkrumah University of Science and Technology (KNUST), Kumasi, Ghana; 3 Department of Parasitology and Microbiology, Centre for Research in Infectious Diseases, Yaounde, Cameroon; 4 TILLER ALPHA GmbH, Bonner Talweg 64, 53113 Bonn, Germany; 5 African Center of Excellence in Bioinformatics and Data Sciences of Mali (ACE-B), University of Sciences Techniques, and Technologies of Bamako (USTTB), Bamako, Mali; 6 Faculty of Pharmacy, Alexandria University, Alexandria, Egypt; 7 Arboviruses and Hemorrhagic Fever Viruses Unit, Virology Department, Institut Pasteur de Dakar, Dakar, Senegal

**Keywords:** Structural biology, Protein structure predictions, Developing world

## Abstract

Without addressing the lack of research infrastructure, funding, and supportive science policies, structural biology capacity-building efforts in Africa will continue to be hindered by the persistent challenge of brain drain.

The 2024 Nobel Prize in Chemistry highlighted once again the transformative role of structural biology in modern science. It recognized the contributions of David Baker to protein design and the development of AlphaFold, led by Demis Hassabis and John Jumper from Google DeepMind. AlphaFold is a state-of-the-art AI tool for predicting protein’s 3D structure from primary amino acid sequence. It accelerates and refines protein modeling essential for structure-based drug design, thus potentially addressing global health challenges.

But AlphaFold represents more than a tool for protein structure prediction; it also contributes to reshaping the paradigms of scientific collaboration and knowledge production. By democratizing access to accurate protein models, it transcends traditional barriers, enabling a broader spectrum of researchers to engage with complex biological problems. For example, researchers from African countries tackling urgent health challenges such as malaria, tuberculosis, HIV/AIDS, Lassa fever, and antimicrobial resistance- now have access to advanced computational tools, previously out of reach. This democratization empowers scientists working on critical, region-specific health issues and exemplifies how technological innovations can influence both scientific discovery and the social frameworks of science, fostering inclusivity and accelerating progress in global health research.

AlphaFold^1,2^ holds significant promise for advancing structural biology in Africa, where pressing health challenges require cutting-edge technologies. By offering a freely accessible platform, AlphaFold helps bridge resource gaps, democratizes access to advanced technologies and empowers scientists to drive critical discoveries. However, as seen in initiatives that integrate AlphaFold into training programs for scientists in these regions, technology alone is not enough to elevate structural biology research in African countries. Comprehensive support, including mentorship, infrastructure, and funding, is essential to fully realize its potential^3^.

The BioStruct-Africa initiative, a not-for-profit organization founded in 2017 and registered in both Sweden and Ghana, is dedicated to developing structural biology expertise across Africa^4,5^. The organization provides cutting-edge training in advanced techniques, including cryo-electron microscopy (cryo-EM) and X-ray crystallography, complemented by post-workshop online mentoring to ensure long-term skill retention and sustainable impact. As part of its mission, BioStruct-Africa has integrated AlphaFold into its training programs to advance the field of structural biology on the continent. Recently, for the fifth time, it conducted a workshop where early career researchers and students from across the continent gained essential skills in using AlphaFold. The workshop was in very high demand. From a highly competitive pool of over 100 applicants, 20 participants were selected through a rigorous process that prioritized diversity and ensured balanced gender representation. Nearly all of them found AlphaFold highly relevant to their current research and considered it a valuable addition to their skill set. The training quality was also highly rated, with most attendees expressing strong satisfaction. Feedback indicates that participants felt well-prepared to apply their new skills to ongoing research and to teach others—an important step toward BioStruct-Africa’s goal of building a community of structural biologists in Africa and fostering collaboration across the continent.

While gains in skills and knowledge during the workshop align with BioStruct-Africa’s mission to build capacity and improve expertise in structural biology, one key issue emerged from the feedback: participants’ intentions to relocate to the Global North for further studies or career advancement as well as the quest for more practical training in laboratories or at larger research infrastructures.

A substantial majority of participants expressed a strong interest in relocating to countries in the Global North for extended periods, with half already taking concrete steps toward this goal. Many intended to remain abroad long-term, primarily to pursue advanced degrees such as master’s or doctoral programs. When asked about the factors driving this trend, participants cited significant deficiencies in research infrastructure, inadequate funding, and limited access to practical training opportunities in their home countries. These findings underscore two critical issues: the lack of opportunities for early career researchers and students to pursue master’s or doctoral-level training, and the challenge of retaining researchers in Africa, who, once they leave the continent, often do not return.

Participants emphasized that increased investment in laboratories, equipment, and training could bridge the gap between theoretical knowledge and practical application, making it more feasible for them to work locally. They also stressed the importance of stable employment, collaborative networks, and a supportive environment with access to quality education, healthcare, and family resources. Many believed that these improvements would create a more competitive and secure work environment, reducing the drive to seek opportunities abroad—a trend that risks depleting Africa’s scientific expertise. African researchers who settle abroad often face challenges returning due to limited funding and infrastructure back home. Furthermore, those who remain abroad may realign their research priorities with those of their host institutions, diverting attention from Africa’s pressing health challenges. This cycle not only disrupts progress but also weakens essential training and mentorship networks necessary for building sustainable local research infrastructure and community. These findings highlight two key challenges: the limited availability of training opportunities for early career researchers and students, and the difficulty of attracting back researchers who frequently choose to remain abroad after relocating for initial or advanced training.

At the same time, workshop participants also highlighted their commitment to local capacity building. The majority indicated a strong likelihood of recommending the workshop to their peers, suggesting a desire to contribute to a thriving community of structural biology practitioners within Africa. This commitment to peer engagement is a promising sign that, with the right resources, many of these scientists would prefer to stay and contribute to research that directly impacts their communities.

This feedback underlines the gap between technological opportunities on the one hand and favorable conditions in terms of science policy and infrastructure on the other. The BioStruct-Africa’s workshops on AlphaFold and the mentoring programs are a valuable component of capacity-building, but it is only one part of a broader ecosystem necessary for scientific innovation. To counter the highlighted challenges and retain talent, it is crucial to address the infrastructure and resource gaps that lead scientists to seek opportunities overseas. Investments in basic research infrastructure are essential to create an environment where African scientists can conduct high-quality research.

The field of science policy aimed at fostering better local opportunities is undoubtedly evolving and gaining momentum. Initiatives like the African Bioinformatics Institute, which was established in October 2024 supported by the Wellcome Trust, and the Chan Zuckerberg Initiative are beginning to address some of these needs. Institutes and initiatives like these can lay the groundwork for a more self-sufficient research ecosystem among African countries. The BioStruct-Africa AlphaFold workshop is a successful example of the immediate impact that high-quality training can have on local researchers’ skills and confidence. However, the broader issue of brain drain underscores the need for systemic changes to support the long-term retention of trained scientists.

The integration of advanced technologies, grassroots initiatives, and emerging scientific communities, supported by complementary science policy efforts, should work in unison to create a research ecosystem that would enable scientists to thrive within their local and regional contexts while maintaining strong connections to their international peers. To truly leverage the potential of programs like BioStruct-Africa, countries in Africa and global stakeholders must collaborate to support initiatives that align with Africa’s unique health needs. Then it is possible to not only retain talent but also catalyze a new era of innovation and health research rooted in Africa and embedded in global science.
